# Proteins and Peptides from Food Sources with Effect on Satiety and Their Role as Anti-Obesity Agents: A Narrative Review

**DOI:** 10.3390/nu16203560

**Published:** 2024-10-20

**Authors:** Anaís Ignot-Gutiérrez, Gloricel Serena-Romero, Daniel Guajardo-Flores, Mayvi Alvarado-Olivarez, Armando J. Martínez, Elvia Cruz-Huerta

**Affiliations:** 1 Instituto de Neuroetología, Universidad Veracruzana, Av. Dr. Luis Castelazo Ayala s/n, Industrial Ánimas, Xalapa 91193, Veracruz, Mexico; zs20022738@estudiantes.uv.mx (A.I.-G.); malvarado@uv.mx (M.A.-O.); 2 Centro de Investigaciones Biomédicas, Universidad Veracruzana, Av. Dr. Luis Castelazo Ayala s/n, Industrial Ánimas, Xalapa 91193, Veracruz, Mexico; zs20000316@estudiantes.uv.mx; 3 Tecnológico de Monterrey, Escuela de Ingeniería y Ciencias, Centro de Biotecnología FEMSA, Eugenio Garza Sada 2501 Sur, Monterrey 64849, Nuevo León, Mexico; danielgdo@tec.mx; 4 Centro de Investigación y Desarrollo en Alimentos, Universidad Veracruzana, Av. Dr. Luis Castelazo Ayala s/n, Industrial Ánimas, Xalapa-Enríquez 91193, Veracruz, Mexico

**Keywords:** dietary proteins, bioactive peptides, gut hormones, appetite, satiety, obesity

## Abstract

Background/Objective: Obesity, clinically defined as a body mass index (BMI) of 30 kg/m^2^ or higher, is a medical condition characterized by the excessive accumulation of body fat, which can lead to adverse health consequences. As a global public health issue with an escalating prevalence, controlling appetite and satiety is essential for regulating energy balance and managing body weight. Dietary proteins and peptides have gained interest in their potential to prevent and treat obesity by modulating satiety signals. This narrative review analyzes scientific evidence highlighting the role of dietary proteins and peptides in regulating satiety signals and investigates their therapeutic potential in preventing and treating obesity. Methods: A comprehensive literature search was conducted in multiple electronic databases, including PubMed, Scopus, and Web of Science. The search focused on articles examining the impact of dietary proteins and peptides on satiety and obesity, encompassing both preclinical and clinical trials. Results: Several studies have demonstrated a correlation between the intake of specific proteins or peptides from plant and animal sources and satiety regulation. These investigations identified mechanisms where amino acids and peptides interact with enteroendocrine cell receptors, activating intracellular signaling cascades that promote the release of anorexigenic gut hormones such as cholecystokinin (CCK), glucagon-like peptide-1 (GLP-1), and peptide YY (PYY). Both in vitro and in vivo assays have shown that these interactions contribute to appetite regulation and the sensation of satiety. Conclusions: Using proteins and peptides in the diet may be an effective strategy for regulating appetite and controlling body weight. However, more research—including clinical trials—is needed to understand the underlying mechanisms better and optimize the application of these bioactive compounds in preventing and treating obesity.

## 1. Introduction

Obesity is a clinical condition defined by the excessive accumulation of adipose tissue in the body, characterized by a body mass index (BMI) of 30 kg/m^2^ or higher [1]. This condition arises from complex genetic, socioeconomic, and cultural interactions [2]. In addition, consumption habits, urban development, and lifestyle significantly impact the incidence of obesity, which is recognized as a relevant risk factor for the development of various comorbid diseases [1]. In recent decades, the prevalence of obesity worldwide has grown substantially. Currently, it affects more than 700 million people globally, and it is estimated that by 2030, there will be 1.35 billion overweight adults and 573 million obese adults [3]. Central and peripheral hormones regulate food intake and energy balance, with neuropeptides acting in multiple brain areas and peripheral organs to form an integrated gut–brain axis [4]. Therefore, regulating satiety through hormone modulation may reduce excessive energy intake and prevent overweight and obesity.

Satiety is a physiological process indicating the sensation of fullness related to controlling hunger and appetite. Food intake produces satiety through mechanical stimulation [5]. In turn, gastrointestinal peptides are secreted, sending signals to the hypothalamus and stimulating hormone secretion in enteroendocrine cells [6]. The main satiety-inducing hormones are cholecystokinin (CCK), glucagon-like peptide-1 (GLP-1), and peptide YY (PYY) [7].

Proteins are the most effective macronutrient for decreasing or inhibiting food intake compared to lipids and carbohydrates [8,9,10]. As they pass through the gastrointestinal tract, enzymes hydrolyze proteins, releasing a diversity of peptides and free amino acids [11], which induce the secretion of satiety-related hormones in enteroendocrine cells [12,13]. Additionally, proteins stimulate energy expenditure by increasing thermogenesis and may act directly on areas of the hypothalamus that regulate food intake [9]. Several in vitro and in vivo studies have reported a direct relationship between the source of proteins and their ability to induce satiety [14,15], causing different responses in inducing the secretion of one or several anorexigenic hormones [14]. Notably, whey proteins from milk have demonstrated a superior satiating effect than other food sources [16].

In recent years, significant interest has arisen in studying bioactive peptides derived from dietary proteins. Bioactive peptides are sequences of amino acids from proteins linked by peptide bonds, which can be released by in vitro hydrolysis, in vivo gastrointestinal digestion, or microbial fermentation. The size of these peptides can vary from 2 to 30 amino acid residues, and they can exert different biological activities, including antihypertensive, antioxidant, antimicrobial, antidiabetic, immunomodulatory, and anticancer properties, depending on their amino acid composition, sequence, and structure [17,18]. Additionally, some bioactive peptides can prevent fat accumulation and regulate appetite and energy metabolism, positioning them as potentially effective agents in the fight against obesity [19].

Peptides and amino acids derived from dietary proteins act through interaction with membrane receptors of enteroendocrine cells [9,20], which are nutrient-sensitive, including amino acids [21]. These receptors are predominantly G protein-coupled receptors (GPCRs), such as taste receptor type 1 (T1R), G-protein coupled receptor 39 (GPR39), G protein-coupled receptor family C group 6 member A (GPRC6A), and the calcium-sensing receptor (CaSR) [22,23].

It is important to note that the effects of dietary proteins and peptides on satiety and appetite regulation can vary significantly among individuals. Factors such as genetic variability, age, sex, metabolic status, and gut microbiota composition can all influence these effects. Differences in digestion and metabolism rates and individual metabolic responses can also impact the efficacy of proteins and peptides in modulating satiety signals. Additionally, other macronutrients, food matrix effects, and meal patterns can affect the bioavailability and activity of these bioactive compounds [24]. Therefore, while dietary proteins and peptides show promise as functional foods for obesity management, it is crucial to consider these differences when developing dietary recommendations.

In this context, this review presents the current state of research demonstrating the role of dietary proteins in inducing satiety, highlights specific peptide sequences derived from protein digestion, and explores their impact on satiety hormone stimulation. The insights garnered could provide a basis for developing strategies to identify and isolate peptide sequences with secretagogue effects and contribute to structure–function assessments necessary for conducting future clinical trials on appetite control. Such strategies may be considered for preventing and controlling obesity and contributing to public health.

## 2. Methods

A narrative review search strategy was used to identify relevant literature on the impact of food-derived proteins and peptides on satiety regulation and appetite control, essential factors in preventing and treating obesity, based on the scale for the quality assessment of narrative review articles (SANRA) [25]. An extensive search of electronic databases, including PubMed, Web of Science, Scopus, and Science Direct, using keywords such as “dietary proteins”, “peptides”, “anorexigenic gastrointestinal peptides”, “satiety”, “appetite”, and “obesity”. Furthermore, bibliographies of the identified manuscripts were reviewed to identify additional pertinent articles and other relevant data sources applicable to this review. Inclusion criteria were limited to research articles and reviews published in English between 2000 and 2024 that directly examined both in vitro and in vivo (in animals and humans) the effects of intact proteins, hydrolysates, and peptides of animal and plant origin on anorexigenic hormone secretion, food intake, and satiety. Exclusion criteria ruled out articles in languages other than English, studies published before 2000, and studies working with bioactive compounds other than proteins to ensure consistency and relevance of the data analyzed. Abstracts and full text were reviewed, culminating in a final selection of 86 articles. This selection facilitated the compilation of a cohesive narrative that shows how proteins and food-derived peptides modulate the satiety response.

## 3. Gut Hormones Related to Satiety

**Figure 1:**
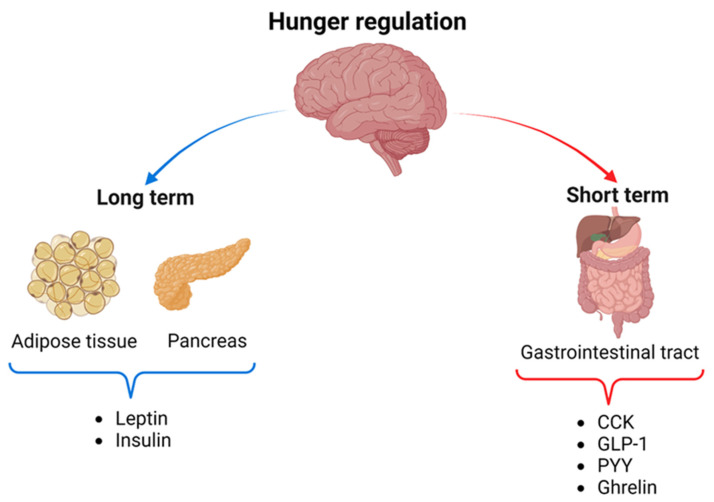
Short- and long-term mechanisms of hunger regulation. In the long term, adipose tissue releases leptin, and the pancreas releases insulin, which influences the hypothalamus to regulate appetite and metabolism sustainably. In the short term, the gastrointestinal tract releases hormones such as cholecystokinin (CCK), glucagon-like peptide type 1 (GLP-1), tyrosine-tyrosine peptide (PYY), and ghrelin in response to food intake, rapidly interacting with the brain to regulate feelings of hunger and satiety after eating [39]. Created in BioRender.

The gut–brain axis is a bidirectional communication pathway connecting the gut and brain, using hormonal and neural signals to regulate metabolic homeostasis [26]. During food intake, the gut sends signals to the central nervous system, particularly the hypothalamus, providing information about the size and composition of the meal [27]. The hypothalamus plays a central role in controlling energy balance and regulating satiety by processing signals from hormones such as CCK, GLP-1, PYY, insulin, and leptin, which promote the sensation of fullness [28] (Figure 1).

CCK, an intestinal peptide hormone, is synthesized and secreted by I cells in the cranial part of the small intestine in response to the intake of proteins or their hydrolysates [29,30]. This peptide circulates in the blood after food ingestion and acts as a neurotransmitter in central nervous system neurons in the brain. Also, CCK exerts its anorexigenic effect through receptors on the vagus nerve and peripheral nerves innervating the gastrointestinal tract, particularly in the intestines [31]. Furthermore, it regulates anthropyloroduodenal motility, modulates pancreatic protein secretion and gallbladder contractility, delays gastric emptying, suppresses appetite through CCK receptors on the vagus nerve, and induces satiety through low-affinity receptors that signal to the brain [32,33,34].

GLP-1 is a proglucagon derivative secreted by L cells located mainly in the ileum and colon. GLP-1 is an incretin hormone released after food intake and in response to nutrients such as glucose, carbohydrates, proteins, and fats [5,35]. Furthermore, this hormone plays multiple roles: it increases glucose-dependent insulin secretion and inhibits glucagon release by the pancreas. Moreover, GLP-1 slows gastric emptying and regulates satiety and food intake by signaling through the vagus nerve [36,37,38].

PYY is a hormone that, like GLP-1, is synthesized in L cells throughout the distal small intestine and colon. It influences food intake and satiety by activating G-protein-coupled Y2 receptors (Y2R) on neuropeptide Y (NPY) and Agouti-related peptide (AgRP) neurons in the arcuate nucleus of the hypothalamus. This activation triggers a signaling cascade that suppresses appetite-stimulating NPY neurons, allowing disinhibition of the proopiomelanocortin/α-Melanocyte-stimulating hormone (POMC/α-MSH) pathway, which induces satiety [40].

## 4. Dietary Proteins as a Source of Bioactive Peptides with Satiety Effects

**Figure 2:**
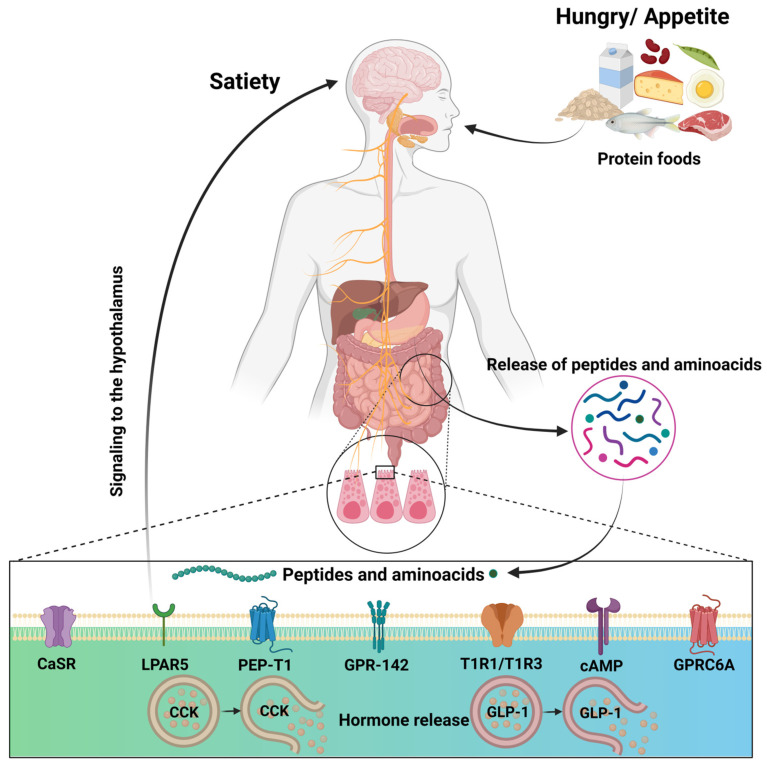
Representative scheme of appetite and satiety regulation by dietary proteins. Shows how protein-rich foods influence the regulation of appetite and satiety by releasing peptides and amino acids. These compounds interact with various receptors on enteroendocrine cells in the gut, stimulating the release of anorectic hormones that signal the brain to control appetite [41]. Created in BioRender.

The endoendocrine cells detect the luminal content at the intestinal level using specific nutrient receptors. These cells secrete hormones such as CCK and GLP-1, which are critical for energy homeostasis because they inhibit gastric emptying and reduce gastric acid secretion [5]. During protein digestion, peptides and amino acids are released, which influence the secretion of these hormones. Figure 2 shows that peptides and amino acids released during digestion interact with receptors on neuroendocrine cells, stimulating the secretion of anorectic hormones such as CCK and GLP-1, which send signals to the brain to regulate appetite, contributing to satiety and reducing food intake.

## 5. Mechanisms of Peptides and Amino Acids in the Secretion of Anorexigenic Hormones

Amino acids and peptides induce the secretion of anorexigenic hormones in entero-endocrine cells through various mechanisms, primarily via interaction with cell membrane receptors. In vitro studies have assessed the secretagogue effect of dietary proteins and peptides on STC-1 and GLUTag cell lines, focusing mainly on the secretion of the hormones CCK and GLP-1 [21]. The receptors associated with the interaction of amino acids and peptides include G protein-coupled receptors (GPR), calcium-sensing receptors (CaSR), amino acid taste receptors (T1R1/T1R3), and peptide transporters (PEPT) [11]. However, some peptides and amino acids also stimulate hormone secretion by activating cyclic adenosine monophosphate (cAMP) and extracellularly regulated kinase (ERK1/2) signaling pathways [42].

**Figure 3:**
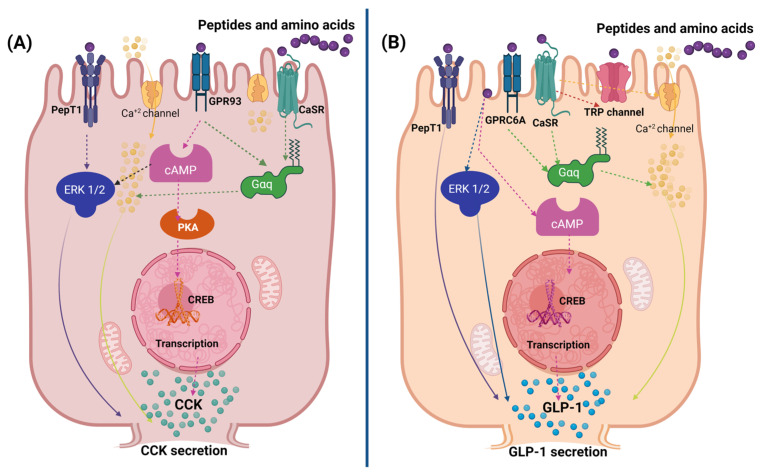
Possible mechanisms by which amino acids and peptides induce the secretion of anorexigenic hormones in enteroendocrine cells, specifically the secretion of CCK and GLP-1. (**A**) CCK secretion. Amino acids and peptides interact with several cell membrane receptors in enteroendocrine cells, including peptide transporters (PepT1), calcium (Ca^2+^) channels, G protein-coupled receptors (GPR93), and calcium-sensitive receptors (CaSR). These interactions can activate several intracellular signaling pathways: ERK1/2 pathway: Interaction of peptides and amino acids with PepT1 and calcium channels can activate the ERK1/2 pathway, leading to gene transcription and CCK secretion. cAMP pathway: activation of GPR93 and CaSR can increase intracellular cAMP levels, activating protein kinase A (PKA) and leading to CREB phosphorylation, promoting gene transcription and CCK secretion. (**B**) GLP-1 secretion. Amino acids and peptides induce GLP-1 secretion by interacting with several receptors: Peptide Transporters (PepT1) Stimulate the ERK1/2 pathway. GPRC6A and CaSR receptors activate the cAMP pathway and CREB signaling. Calcium channels and TRP channels: They participate in the increase of intracellular Ca^2+^ levels, which triggers intracellular signaling cascades [46]. Created in BioRender.

Amino acids and peptides that activate CaSR and GPR receptors increase intracellular Ca^2+^ levels, triggering the calcium cascade and initiating the transcription of genes and the secretion of hormones [43]. In cases where calcium levels do not increase but hormone secretion is stimulated, it has been proposed that this occurs through the activation of the cAMP pathway and the transcription of proglucagon or through the activation of the intracellular ERK1/2 and mitogen-activated protein kinase (MAPK) pathways, causing hormone exocytosis [15,44]. Interestingly, hormone release can also occur without activating these pathways through direct permeation of the cell membranes [45] (Figure 3).

Specifically, GLP-1 is primarily stimulated by dipeptides and tripeptides, and certain free amino acids such as phenylalanine (Phe), valine (Val), tryptophan (Trp), and lysine (Lys) through GPR93 (also known as lysophosphatidic acid receptor 5, LPAR5) and PEP-T1 receptors [47]. On the other hand, CCK is stimulated by free amino acids such as phenylalanine (Phe), leucine (Leu), and glutamate (Glu), as well as by peptides with one or two glutamic residues like Glu-Glu-Phe/-Met/-Val through the activation of CaSR, T1R1–T1R3, and GPR93 [48,49,50].

Some investigations with different dietary proteins have shown that peptides containing aromatic amino acids enhance CCK secretion, highlighting the importance of specific amino acid residues in this process [48]. In contrast, Tulipano et al. [51] reported that peptide length influences CCK release more than the amino acid sequence and that peptides of five or more amino acids are required for optimal hormone secretion. Similarly, Komatsu et al. [45] indicated that a highly hydrophobic peptide sequence effectively induces GLP-1 secretion in GLUTag cells. Furthermore, Santos-Hernández et al. [11] reported that CCK secretion in STC-1 cells was triggered by peptides that had between 4 and 11 amino acids of varying length and hydrophobicity. However, GLP-1 secretion was mainly induced by peptides with high hydrophobicity.

**Figure 4:**
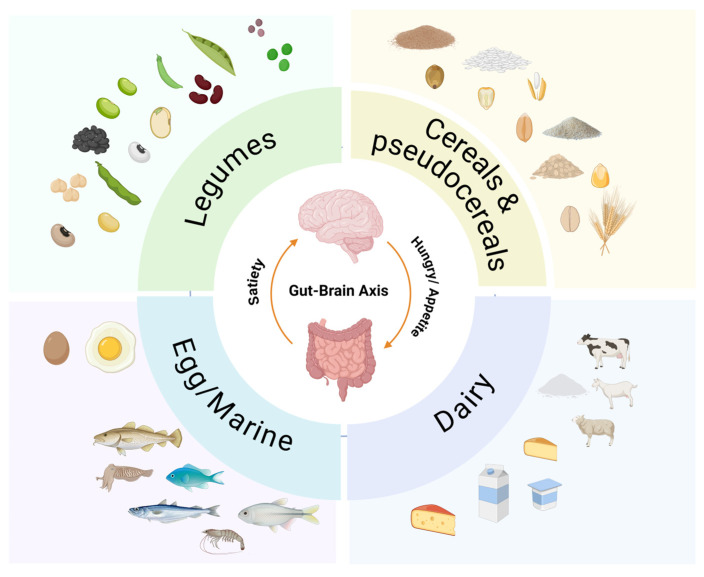
Protein sources from animal and plant origins with their potential impact on satiety [52]. Created in BioRender.

The impact of some dietary proteins, both of animal and plant origin, on satiation regulation has been investigated. However, studies have focused mainly on specific sources of proteins as precursors to bioactive peptides, mainly from legumes, cereals, dairy products, egg whites, and some marine proteins, as illustrated in Figure 4.

## 6. Plant Proteins and Peptides on the Hormonal Regulation of Satiety: In Vitro and In Vivo Studies

Research has highlighted the ability of some plant proteins, their hydrolysates, and peptides to stimulate satiety through the secretion of anorexigenic hormones. This effect has been observed in both in vitro and in vivo studies, and various plant protein sources have demonstrated a potential impact on satiety. In vitro studies have provided a controlled environment to investigate the specific mechanisms through which plant proteins and peptides influence hormone secretion and satiety (Table 1). Some in vivo studies have been extended to more complex biological systems, including animal and human models (Table 2 and Table 3).

In particular, the tripeptide Arg-Ile-Tyr (RIY), isolated from rapeseed, has reduced food intake and gastric emptying in male ddY mice. This effect has been associated with CCK secretion through the CCK1 receptor, as demonstrated by blocking this effect with a CCK1 receptor antagonist [53]. Similarly, the peptide sequence VRIRLLQRFNKRS corresponding to the active fragment 51–63 of soybean β-conglycinin increased portal plasma CCK concentration and reduced food intake in male Sprague-Dawley rats. This result was obtained by intraduodenal administration of pepsin-hydrolyzed soybean β-conglycinin, showing superior efficacy compared with intact protein, although no specific receptors were identified [54]. Furthermore, studies with mouse enteroendocrine STC-1 cells [29] evaluated extracts of field beans (*Dolichos lablab*) and Yard long beans (*Vigna sesquipedalis*) and demonstrated the ability of a 51 kDa globular protein, termed dolicholine, to increase CCK secretion up to 2.5 times more than β-conglycinin.

This underscores the rich diversity of plant sources regarding hormonal impact, providing a wide range of potential research avenues [54]. Among legumes, peas have shown satiating effects. For example, Geraedts et al. [55] demonstrated that intact proteins from peas (*Pisum sativum*) (NUTRALYS^®^) and wheat effectively stimulated CCK and GLP-1 secretion in STC-1 cells. However, their hydrolysates did not show this effect, probably due to the type of enzymatic hydrolysis used (subtilisin). Furthermore, in male SPF Wistar rats, NUTRALYS^®^ pea proteins increased plasma levels of CCK, GLP-1, and PYY, with an effect comparable to that of whey protein [56].

On the other hand, the study of cereal and pseudocereal proteins such as quinoa and amaranth has also contributed to understanding their satiating bioactivity. These studies have shown that the effects can reduce food intake and body mass in rats, with amaranth generating a higher concentration of CCK and leptin and reducing ghrelin, while quinoa only increases CCK [57]. In contrast, a low molecular weight wheat hydrolysate, composed mainly of di-, tri-, and tetrapeptides obtained from bacterial protease, increased GLP-1 secretion in GLUTag cells and Sprague-Dawley rats after intraperitoneal injection of glucose. This bioactivity activates the Ca^2+/^calmodulin-kinase II-dependent pathway [58]. On the other hand, in a study with Landrace Yorkshire sows, feeding wheat aleurone increased serum PYY and GLP-1 levels, improving postprandial satiety [59].

Recent studies on barley-derived protein hydrolysates have further expanded this field of research. Huang et al. [60] demonstrated that PDLP, YRIVPL, and VFLQPH peptide sequences of barley proteins stimulated CCK secretion in STC-1 cells. These findings were confirmed in vivo, where oral administration of the hydrolysates to male ICR mice increased plasma CCK levels. Similarly, Song et al. [61] identified a novel peptide (RYIVPL) released after the simulated gastrointestinal digestion of wheat protein that significantly stimulated CCK secretion in vitro using STC-1 cells. This effect was confirmed in vivo, with male ICR mice showing increased plasma CCK levels after oral administration of wheat protein hydrolysate. Furthermore, the study revealed that the calcium-sensing receptor (CaSR) plays a crucial role in CCK secretion induced by digesting wheat protein containing the RYIVPL peptide.

These studies highlight plant-derived proteins and peptides’ enormous potential in regulating satiety hormones. However, their efficacy and mechanisms may vary depending on the protein source, processing methods, and specific peptides. Standardized protocols and further studies are needed to corroborate such effects.

**Table 1:** In vitro studies on the effects of plant-derived proteins and peptides in cellular models.

Protein Source		Peptide Sequence	Study Model	Dose	Biological Activities	Reference
Legumes	Country bean (*Dolichos lablab*)	Hydrolysate (Pepsin and pronase)	STC-1 cells	5 mg/mL solid or protein weight	Stimulates CCK secretion	[29]
	Yard long bean (*Vigna sesquipedalis*)	Hydrolysate (Pepsin and pronase)	STC-1 cells	5 mg/mL solid or protein weight	Stimulates CCK secretion	[29]
	Pea	Hydrolysate (Subtilisin)	STC-1 cells	1 mg/mL of protein	Stimulates CCK secretion	[55]
	Soybean	Hydrolysate (commercial)	STC-1 cells and GLUTag cells	5–10 mg/mL	Stimulates CCK and GLP-1 secretion	[7]
Cereals	Maize/zein	Hydrolysate (Papain)	GLUTag cells	5 mg/mL of hydrolysate	Stimulates GLP-1 secretion	[62]
	Wheat	Hydrolysate (Papain)	STC-1 cells	5–20 mg/mL of hydrolysate	Stimulates GLP-1 secretion	[55]
	Wheat	Hydrolysate (Bacterial protease)	STC-1 cells and GLUTag cells	1 mg/mL of hydrolysate	Stimulates GLP-1 secretion	[58]
	Wheat/gluten	Hydrolysates (commercial)	STC-1 cells	5 and 10 mg/mL of hydrolysate	Stimulates CCK and GLP-1 secretion	[58]
	Highland barley (*Hordeum vulgare* L.)	Protein digested (In vitro)	STC-1 cells	5 mg/mL of digested	Stimulates CCK secretion	[60]
	Wheat	Protein digested (In vitro)	STC-1 cells	5 mg/mL of digested	Stimulates CCK secretion	[61]

**Table 2:** In vivo studies on the effects of plant-derived proteins and peptides in animal models.

Protein Source	Proteins/Peptides	Study Model	Assessment	Dose	Biological Activities	Reference
Legumes	Soybean, Péptida, β 51–63 (VRIRLLQRFNKRS)	Male Sprague-Dawley rats	Intraduodenal infusion	7.5 nmol = 2.5 mL of 3 μmol/L	Suppresses food intake via CCK release	[54]
	Pea, Hydrolysates (Pepsin)	Male SPF Wistar rats	Orally administered	Isocaloric meals containing 35% energy of pea-protein	Stimulates CCK, GLP-1, and PYY secretion	[56]
	Rapeseed, Tripeptide Arg-Ile-Tyr (RIY)	Male ddY mice	Intraperitoneally administered	50 mg/kg body weight	Stimulates CCK secretion	[53]
Cereals	Maize/zein, Hydrolysate (Papain)	Sprague-Dawley rats	Ileal administration, blood samples 15, 30, and 60 min after oral administration	500 mg/2 mL of water	Stimulates GLP-1 secretion	[63]
	Maize/zein, Hydrolysate (Papain)	Sprague-Dawley and Goto Kakizaki rats	Orally administered, blood samples 15, 30, 60, 90, and 120 min after oral administration	2 g/kg body weight	Stimulates GLP-1 secretion	[64]
	Rice, Hydrolysates (Papain and pepsin)	Male Sprague-Dawley rats	Orally administered, blood samples 15, 30, 60, 90, and 120 min after oral administration	2 g/kg body weight	Increases GLP-1 secretion and decreases plasma DPP-IV activity	[65]
	Highland barley (*Hordeum vulgare* L.)	Male ICR mice	Oral gavage, blood samples 0, 15, 30, 60, 90, 120, and 150 min	1 g/kg body weight	Stimulates CCK secretion	[60]
	Wheat, Hydrolysate (Bacterial protease)	Sprague-Dawley rats	Orally administered, blood samples 15, 30, and 60 min after oral administration	2 g/kg body weight	Stimulates GLP-1 secretion	[58]
	Wheat, Hydrolysate (commercial)	Male Wistar/ST rats	Orally administered, blood samples 1, 2, 3, and 6 h after oral administration	1 g/kg body weight	Stimulates CCK, GLP-1, and PYY secretion	[7]
	Wheat, Hydrolysate	Landrace Yorkshire sows	Orally administered, blood samples on days 70 and 109 of gestation, day 0 of lactation, and the day of weaning	15% of total dietary constituents	Increases plasma PYY and GLP-1 concentrations	[59]
	Wheat protein	Male ICR mice	Oral gavage, blood samples at 15, 30, 60, 90, 120, and 150 min	1 g/kg body weight (mice)	Stimulates CCK secretion	[61]
Pseudocereals	Quinoa and amaranth, Quinoa flour and amaranth flour	Male rats of the Wistar albino strain	Orally administered, Blood samples obtained at the end of 15 days	20% of total dietary constituents	Stimulates CCK secretion and amaranth to a greater extent	[57]

**Table 3:** Human studies on the effects of plant-derived proteins and peptides on food intake, satiety, and hormone levels.

Protein Source		Participant (n)	Study	Assessment	Dose	Duration	Biological Activities	Reference
Legumes	Pea	32 males, 20–35 years	Single-blind, randomized, crossover	Visual Analogue Scales at 10 min intervals after preload	20 g of pea protein	1 day: 30 min before and after ad libitum meal for 2 h	Food intake was lower, and satiety was higher	[66]
	Pea	10 lean males, mean age of 25 years, and 10 obese males, mean age of 41 years	Single-blind, randomized controlled crossover	Nasodudenal catheter, blood samples	250 mg/kg body weight	4 weeks: 1 experiment per week	Reduced food intake for both lean and obese subjects and CCK levels increased at 10 and 20 min after protein administration in obese subjects	[67]
	Yellow pea	20 male, 20–30 years	Single-blind, randomized	Visual Analogue Scales for motivation to eat	20 g of yellow pea protein	1 week	Food intake was lower after 30 min, and post-meal blood glucose was suppressed compared to the control	[68]
	Pea	22 females and 11 males, 18–65 years	Double-blind, randomized, placebo-controlled, crossover	Visual Analogue Scales, blood samples	15 g and 30 g of pea protein isolate (NUTRALYS^®^)	4 weeks	Reduced caloric intake. Thirty grams of pea protein led to an increase in perceived levels of satiety	[69]
Cereals	Wheat	27 moderately overweight males with a mean age of 25 years	A single-blinded crossover study	Visual Analogue Scales, blood samples	Wheat protein hydrolysate (2 g) + L-arginine (3.2 g)	1 day: after an overnight fast	Reduced caloric intake. Increases plasma serotonin levels	[70]

## 7. Animal Proteins and Peptides on the Hormonal Regulation of Satiety: In Vitro and In Vivo Studies

Research on animal proteins, hydrolysates, and peptides has revealed their ability to stimulate satiety through the secretion of anorexigenic hormones (Table 4). To this end, it shows an overview of these pioneering studies, covering both in vitro and in vivo investigations. These studies highlight their relevance in nutrition, biochemistry, and obesity treatment and prevention. For instance, Geraedts et al. [55] evaluated the secretagogue effect of different dietary proteins, such as casein, whey protein, cod protein, and egg protein, as well as some of their hydrolysates and synthetic peptides, in STC-1 cells. The authors determined that intact proteins affect satiety hormone secretion substantially. Specifically, casein and whey showed more potent effects on CCK release, whereas casein, cod, and egg did so on GLP-1 release. Egg hydrolysate stimulated CCK and GLP-1 release, whereas the other hydrolysates and synthetic peptides showed no significant effects. This study concluded that only specific intact or partially digested proteins stimulated hormone release, compared to protein hydrolysates and synthetic peptides.

More recent research by Santos-Hernández et al. [11] investigated the secretagogue effects of CCK and GLP-1 in STC-1 cells, using in vitro digests of egg white. The authors found that CCK secretion was stimulated mainly by peptides and free amino acids, whereas GLP-1 secretion was primarily stimulated by peptides but not by free amino acids. Seven peptides were selected and evaluated according to their effect on the secretion of these hormones. Among them, the decapeptide VLLPDEVSGL and pentapeptide VLLPD significantly improved CCK secretion, while the RVASMASEK and PFL sequences stimulated GLP-1 secretion to a greater extent. These findings suggest that the structural variations among these peptides influence their interaction with different cellular receptors, affecting hormone secretion.

Similarly, Caron et al. [15] identified six peptide sequences from in vitro gastrointestinal digestion of bovine hemoglobin. Three of these peptides (ANVST, TKAVEH, and KAAVT) were associated with GLP-1 secretion; two (TKAVEH and KAAVT) also influenced CCK secretion, and one (VAAA) acted as an inhibitor of DPP-IV. These low-molecular-weight peptides (400 to 700 Da) contain aromatic amino acids, a structural feature that may improve their ability to stimulate hormone secretion.

Also, marine animal proteins have been studied for their satiating capacity. In 2008, Cudennec et al. [71] evaluated muscle protein hydrolysates from blue whiting (*Micromesistius poutassou*) and shrimp (*Penaeus aztecus*) on CCK secretion in STC-1 cells, showing a high stimulatory effect. Small peptides (<1500 Da) showed a greater impact on CCK secretion than those of higher molecular weight, the most potent being those between 1000 and 1500 Da. Subsequently, it was shown that blue whiting muscle hydrolysate increased CCK and GLP-1 secretion in STC-1 cells and reduced food intake in Wistar rats, which was associated with increased plasma concentrations of these hormones. In addition, their prolonged administration decreased body weight gain [72].

Nobile et al. [73] explored the effects of blue whiting muscle hydrolysate, obtained through enzymatic hydrolysis, on body composition and the stimulation of CCK and GLP-1 secretion in mildly overweight subjects. Two doses, 1.4 and 2.8 g, were administered to determine if the effects were dose-dependent, and the subjects’ body weight was also monitored. The results showed that both doses improved body composition and increased the concentrations of CCK and GLP-1 in the participants. Additionally, Cudennec et al. [74] investigated two by-products of cuttlefish (*Sepia officinalis*) viscera hydrolysates and their impact on CCK and GLP-1 secretion in STC-1 cells after simulated gastrointestinal digestion. These hydrolysates demonstrated a significantly greater potential to stimulate the secretion of both hormones compared to intact protein, achieving higher levels in intestinal digests and inhibiting DPP-IV activity. Although the peptide sequences responsible for this bioactivity were not identified, the results were more favorable for hydrolysates containing smaller peptides. In a related study, Wang et al. [75] found that tilapia skin gelatin hydrolysate increased GLP-1 secretion in streptozotocin-induced diabetic rats after 30 days of treatment. This effect was linked to the amino acid content of the hydrolysate, highlighting the importance of bioactive components in the modulation of satiety-related hormones.

The reviewed studies indicate that various marine and fish protein hydrolysates can improve body composition and stimulate the secretion of satiety-related hormones, such as CCK and GLP-1. The findings suggest that both the dose of the hydrolysate and the presence of specific peptide sizes can significantly influence these effects. These results underscore the crucial role of bioactive components in hormonal regulation and suggest potential applications for weight management and metabolic health.

Recent studies have explored the potential of algal proteins, suggesting that they are a viable, nutrient-rich alternative to traditional animal-based proteins. For example, Wu et al. [76] investigated the effects of the consumption of algal biomass and protein concentrates from *Chlorella vulgaris* and *Pyropia seriata* (nori) on postprandial metabolism and satiety. This randomized controlled trial compared the effects of these algae-derived proteins with soy protein concentrate in healthy Chinese men (10 g biomass/algae protein concentrate/day). Both algal biomass and protein concentrate were comparable to soy protein in terms of palatability and satiety regulation. No significant differences were observed in postprandial glucose, insulin, C-peptide, or appetite-related hormones such as GLP-1. However, the study revealed that nori biomass significantly reduced postprandial triglyceride levels, which may suggest its possible lipid-lowering effects.

These results are in agreement with previous studies highlighting the role of marine proteins as modulators of satiety and metabolic responses. However, the variability in responses in different studies suggests the need for further research, as factors such as protein source, processing methods, and individual metabolic differences may influence the efficacy of these proteins. Therefore, while algal proteins show great promise as functional foods for weight management, more long-term studies are needed to better understand their impact on metabolic health and satiety.

**Table 4:** In vitro studies on the effects of animal proteins and peptides in cellular models.

Protein Source	Proteins/Peptides	Study Model	Dose	Biological Activities	Reference
Egg	Egg white hydrolysates (commercial)	STC-1 cells	1 mg/mL	Stimulates CCK and GLP-1 secretion	[55]
	Lysozyme, VAWRRNRCKGTD, WRNRCKGTD, WIRGCRL, IRGCRL, RGCRL	STC-1 cells	0.250 and 1 mM	Stimulates CCK and GLP-1 secretion	[77]
	Ovalbumin, LGAKDSTRT	STC-1 cells	0.250 and 1 mM	Stimulates CCK and GLP-1 secretion	[77]
Marine	Blue whiting muscle (*Micromesistius poutassou*) hydrolysate	STC-1 cells	1% wt/vol	Stimulates CCK secretion	[71]
	Brown shrimp (*Penaeus aztecus*) hydrolysate	STC-1 cells	1% wt/vol	Stimulates CCK secretion	[71]
	Codfish protein hydrolysates	STC-1 cells	1 mg/mL	Stimulates CCK and GLP-1 secretion	[55]
	Blue whiting muscle hydrolysate	STC-1 cells	0.2–1.0% *w*/*v*	Stimulates CCK and GLP-1 secretion	[72]
	Cuttlefish (*Sepia officinalis*) hydrolysate	STC-1 cells	1.3% *w*/*v*	Stimulates CCK and GLP-1 secretion	[74]
	Blue whiting hydrolysate	STC-1 cells	1.0% *w/v* dw	Stimulates GLP-1 secretion	[78]
Others	Bovine hemoglobin, ANVST, TKAVEH, KAAVT	STC-1 cells	1 g/L dry matter	Stimulates GLP-1 secretion	[15]
	Irish cheddar cheeses	STC-1 cells	10 mg/mL	Stimulates GLP-1 secretion	[79]

## 8. Satiety Potential of Dairy Proteins and Peptides

Dairy proteins are a significant source of bioactive peptides capable of inducing satiety by stimulating the secretion of anorexigenic hormones. Approximately 3.3% of milk is composed of proteins, of which 80% are caseins (α s1, α s2, β, and κ) and 20% are whey proteins (β-Lactoglobulin (β-LG), α-Lactalbumin (α-LA), bovine serum albumin (BSA), immunoglobulin, and lactoferrin [4]. The main studies on dairy proteins and peptides that demonstrate effects on satiety are summarized in Table 5.

### 8.1. Caseins

Osborne et al. [49], investigated the effect of β-casomorphin-7 (YPFPGPI) and a dipeptide derived from β-LG on the secretion of CCK and GLP-1 in STC-1 cells, as well as their in vitro transport and hydrolysis in the Caco-2 cell line. The authors found these peptides have a low in vitro transport rate and rapid hydrolysis. Additionally, three peptides derived from β-casomorphin-7 FPGPI, GPI, and YP and one from β-LG were identified, which showed higher transport rates, mainly YP and GPI. The intact β-casomorphin-7 and its metabolite FPGPI induced CCK secretion comparable to 1 mg mL^−1^ of commercial whey hydrolysate, while none of the peptides induced GLP-1 secretion in STC-1 cells.

On the other hand, Gillespie and Green [80] tested whole casein and its fractions (α-casein, β-casein, and κ-casein) in the pGIP/Neo STC-1 enteroendocrine cell line to evaluate the GLP-1 secretagogue effect. The authors found that all caseins stimulated GLP-1 secretion, with intact casein showing the most significant effect, followed by α-casein and β-casein. Additionally, the sodium caseinate hydrolysate LFC25, obtained by chymosin hydrolysis, demonstrated the ability to increase Ca^2+^ release from intracellular stores in STC-1 cells, promoting GLP-1 secretion after 4 h of exposure [35]. The soluble fraction of the hydrolysate, especially peptides smaller than 3 kDa, showed greater bioactivity, with most peptides identified originating from αs1-casein and containing terminal amino acids that can stimulate GLP-1 secretion. In this regard, a comparison was performed between the digestion pattern and effect on GLP-1 stimulation in the GLUTag enteroendocrine cell line of micellar casein concentrate and sodium caseinate, as well as a mixture of the latter with whey protein isolate [45]. The authors found that the micellar concentrate exhibited higher digestibility and bioactivity, achieving greater GLP-1 secretion. Peptides derived from β-casein were more abundant in the micellar concentrate digests, associated with higher bioactivity. In contrast, sodium caseinate did not stimulate GLP-1 in GLUTag cells, although it did in the STC-1 cell line [55]. Also, peptide GPVRGPFPIIV was identified as responsible for inducing GLP-1 secretion, although with lower bioactivity than the micellar concentrate digests. When the effect of intact casein and casein hydrolysate on the intestinal hormonal response in healthy subjects undergoing surgical procedures such as Roux-en-Y gastric bypass and vertical sleeve gastrectomy was evaluated. It was found that direct jejunal infusion of intact casein or hydrolysate increased GLP-1 concentrations at 25 min without significantly impacting hunger and satiety sensations. This suggests that hormonal secretion needs to interact with other stimuli during food intake to control ingestion [81].

Santos-Hernández et al. [82] investigated the secretagogue effect of whey proteins and caseins on CCK and GLP-1 secretion in STC-1 cells using in vitro gastrointestinal digests and human jejunal effluents. Gastric digests of casein and whey stimulated CCK secretion, and intestinal digests significantly had an even greater effect. Interestingly, GLP-1 secretion was induced by gastric digests of casein and intestinal digests of whey, whereas free amino acids had no impact on hormone release. In addition, human jejunal digests also stimulated CCK and GLP-1 secretion, mainly through small peptides derived from casein and whey digests.

Similarly, Vivanco-Maroto et al. [83] evaluated the effects of casein and its hydrolysate on CCK and GLP-1 secretion using a semi-dynamic in vitro gastrointestinal digestion model. They found that gastrointestinal digestion products from casein hydrolysate stimulated CCK and GLP-1 secretion more effectively in STC-1 cells compared to digestion products from intact casein. The results highlight that hydrolyzed proteins, particularly casein hydrolysates, generate a more potent hormonal response than their intact forms. Therefore, processing proteins into smaller peptides could enhance their bioactivity in the gastrointestinal tract.

**Table 5:** In vitro studies on the effects of milk-derived proteins and peptides in cellular models.

Protein Source	Proteins/Peptides	Study Model	Dose	Biological Activities	Reference
Casein	β-casomorphin-7	STC-1 cells	1000, 500, 250, and 125 μM	Stimulates CCK secretion	[49]
	Intact whole casein, α-casein, and β-casein	STC-1 cells	0.3125–10 mg/mL	Stimulates GLP-1 secretion	[80]
	Sodium caseinate (LFC25)	STC-1 cells	10 mg/mL	Stimulates GLP-1 secretion	[35]
	In vitro digests and human jejunal effluents	STC-1 cells	0.250, 1, and 4 mg/mL	Stimulates CCK and GLP-1 secretion	[82]
	β-casein, GPVRGPFPIIV	STC-1 cells	5 mM	Stimulates GLP-1 secretion	[45]
	αs1-casein, RPKHPIKHQGLPG, RYLGYLE, RYLGY, RYLG, KYLGY, RALG	STC-1 cells	0.250 and 1 mM	Stimulates CCK and GLP-1 secretion	[77]
	κ-casein, SRYPS, RPS	STC-1 cells	0.250 and 1 mM	Stimulates CCK and GLP-1 secretion	[77]
	Micellar and hydrolysate casein	STC-1	2 mg/mL	Stimulates CCK and GLP-1 secretion	[83]
Whey	Commercially intact whey protein	STC-1 cells	1 mg/mL	Stimulates GLP-1 secretion	[84]
	α-Lactalbumin	STC-1 cells	10 mg/mL	Stimulates GLP-1 secretion	[85]
	β-Lactoglobulin hydrolysates, ALPMH	STC-1 cells	2 mM	Stimulates CCK secretion	[51]
	Whey protein digests	STC-1 cells	0.250, 1, and 4 mg/mL	Stimulates CCK and GLP-1 secretion	[82]
	Whey from cow, sheep, and goat milk and a mixture thereof	STC-1 cells	1%	Goat serum further stimulates GLP-1 secretion; the mixture better stimulates CCK secretion	[86]
	Bovine serum, β-Lactoglobulin, α-Lactalbumin, and albumin	STC-1 cells	10 mg/mL	α-Lactalbumin stimulates increased GLP-1 secretion	[85]
	β-Lactoglobulin, LIVTQTM, IPAVFK	STC-1 cells	0.250 and 1 mM	Stimulates CCK and GLP-1 secretion	[77]

### 8.2. Whey

Whey proteins constitute approximately 12% of the total solids in whey and around 5% of the total solids in milk. These proteins contain all essential amino acids, especially branched-chain and sulfur-containing ones, and are characterized by high digestibility [87]. In this regard, the CCK secretagogue effect of peptides obtained by pepsin and trypsin digestion of β-LG and hydrolysates of α-LA and β-LG in STC-1 cells. It was found that the peptides ALPMH and PHLMA exhibited the highest bioactivity for CCK secretion, independent of amino acid sequence [51]. Furthermore, Aoki et al. [88] investigated the effect of enzymatic digests of β-LG on ghrelin secretion in MGN3-1 cells, identifying the peptide LIVTQTMKG (lacto gestatin) as an inhibitor of ghrelin secretion, reducing intracellular cAMP levels without affecting Ca^2+^ levels. The effect of intact whey protein and two commercial hydrolysates (DH 32 and DH 45) on GLP-1 stimulation and DPP-IV inhibition in STC-1 cells has also been evaluated [84]. The authors found that only intact whey protein increased GLP-1 concentration, whereas DPP-IV inhibition was more significant in the hydrolysates, especially DH 45. Similarly, Gillespie et al. [85] investigated the bioactive effect of yogurt whey, cheese whey, β-LG, α-LA, BSA, and β-LG hydrolysates in STC-1 pGIP/neo cells. This research found that whey-derived proteins, even at low concentrations, increased cell proliferation, intracellular GLP-1 levels, and GLP-1 secretion. β-LG was identified as the active component responsible for these effects lost upon hydrolysis. Finally, Sánchez-Moya et al. [86] evaluated the secretagogue effect of whey from different species (cow, sheep, goat, and a mixture of the three) on CCK and GLP-1 secretion in STC-1 cells. The authors also found that gastrointestinal digests significantly stimulate hormone secretion more than undigested and fermented samples. Therefore, goat whey showed the best GLP-1 stimulation, while the whey mixture was the most effective for CCK stimulation.

## 9. Protein-Enriched Food Products

Several studies have evaluated the effect of protein-rich foods or protein or peptide-enriched food products on satiety, using both in vitro and in vivo studies in rats and humans. For instance, Ghazzawi and Mustafa [89] conducted a study in which two types of breakfast were tested: one rich in proteins (egg whites, low-fat thick yogurt, low-fat turkey, cucumber, and an apple) and another rich in carbohydrates (a pastry topped with thyme and olive oil, cucumber, and an apple). Blood samples were obtained from rats at 30, 60, and 120 min to assess the hormones CCK, GLP-1, PYY, ghrelin, and appetite sensation. The results showed that the protein-rich breakfast produced higher levels of PYY and a significant increase in GLP-1 compared to the carbohydrate-rich breakfast, which showed constant values in all measurements. Additionally, the protein-rich breakfast decreased hunger and increased satiety to a greater extent. They concluded that a high-protein breakfast has a more significant effect on satiety, mediated by increased concentrations of GLP-1 and PYY.

In another study, Kondrashina et al. [79] evaluated the potential of Irish Cheddar cheeses to stimulate the hormone GLP-1 in rats and the STC-1 cell line through water-soluble extracts, testing different cheese maturation times (2, 4, 6, 8, and 10 months). It was found that Cheddar cheese can stimulate GLP-1 secretion in the cell line, but oral gavage feeding in mice did not show an acute effect on food intake. Furthermore, in vitro studies have demonstrated the loss of bioactivity during intestinal digestion. Studies with young and obese women have shown that there is an effect of an isocaloric drink with whey protein or maltodextrins on satiety and the secretion of the hormones GLP-1 and PYY in young, obese women [9]. It was also reported that whey proteins induced significantly more satiety and less hunger compared to maltodextrins, an effect that began 30 min after ingestion and persisted up to 120 min. Moreover, the intake of each drink significantly increased the levels of GLP-1 and PYY, but this increase was greater with whey proteins.

Finally, in overweight and obese individuals, the effects of biscuits fortified with whey protein isolate and wheat bran on body composition, food intake, appetite, and the secretion of satiety [90]. The study tested four groups: biscuits with whey protein isolate and wheat bran, biscuits with only whey protein isolate, biscuits with only wheat bran, and a control group consumed for eight weeks. Differences between groups in anthropometric parameters, intake, and physical activity level were demonstrated accordingly. Also, body mass, body mass index, and waist circumference decreased in individuals who consumed the whey protein isolate biscuits, alongside a reduction in overall energy intake. Despite these changes, no significant differences were observed in appetite-regulating hormones like ghrelin, leptin, and serotonin. However, GLP-1 levels did increase after consuming whey protein-fortified biscuits.

## 10. Limitations and Future Prospects

Among the limitations identified in the studies selected for this review, the scarce identification and isolation of specific peptide sequences that provide crucial information on which structures are responsible for secretagogue effects and the identification of target molecules stand out. This would allow for the identification of a pattern in the molecular characteristics of bioactive peptides. Additionally, many of the selected studies are in vitro assays which, while informative, require further validation through human studies to determine the effect on dietary intake, raising the need to conduct clinical trials in both men and women, in addition to the lack of evaluation of long-term effects on satiety signals and body composition. Furthermore, as with narrative reviews, the current work may be biased in the selection of studies included in this review.

Despite these limitations, this narrative review provides us with a valuable synthesis of the existing evidence on the role of bioactive proteins and peptides in the regulation of satiety, which can serve as a basis to guide future clinical and experimental trials in appetite control through the secretagogue mechanism of anorexigenic hormones, making them potential candidates for the development of enriched food products or nutraceuticals as a therapeutic strategy for the prevention and management of obesity with lower risks of adverse effects over conventional therapies, which could benefit the food and pharmaceutical industry and contribute to public health.

## 11. Conclusions

The studies reviewed showed that consuming some proteins or peptides of plant or animal origin can influence satiety regulation. Bioactive peptides derived from sources such as soy, pea, quinoa, and milk proteins have shown stimulatory effects on satiety hormone secretion in both in vitro and in vivo studies. In addition, specific mechanisms of interaction between amino acids and peptides with cell membrane receptors have been identified, triggering intracellular signaling cascades that promote the release of these hormones.

Milk proteins, particularly caseins and whey proteins, have been proven highly effective in inducing satiety. Studies have pinpointed specific peptides derived from these proteins that interact with receptors such as GPR, CaSR, and T1R1/T1R3, increasing intracellular Ca^2+^ levels and activating signaling pathways such as cAMP and ERK1/2, culminating in the secretion of anorexigenic hormones. Furthermore, protein-enriched food products, such as protein-rich breakfasts, aged cheeses, and enriched biscuits, have positively affected appetite regulation and body composition.

The evidence reviewed suggests that including dietary protein and its derivatives may be an effective appetite and weight control strategy. However, most studies have been conducted in vitro or controlled animal models, which may only partially reflect the complexity of human physiological responses. Therefore, further research involving human clinical trials is needed, as it is essential to confirm the efficacy of these peptides. In addition to assessing their ability to modulate appetite and body weight, their safety and potential long-term side effects should also be evaluated, providing valuable information on the practical use of dietary peptides to prevent and treat obesity.

## Abbreviations

α-LA	α-Lactalbumin
α-MSH	α-Melanocyte-stimulating hormone
β-LG	β-Lactoglobulin
AgRP	Agouti-related peptide
BMI	Body Mass Index
BSA	Bovine serum albumin
CaSR	Calcium-sensing receptor
CCK	Cholecystokinin
ERK	Extracellularly regulated kinases
GLP-1	Glucagon-like peptide type 1
GPCRs	G protein-coupled receptors
GPR	G protein-coupled receptors
GPR39	G-protein coupled receptor 39
GPR93	G-protein coupled receptor 93
GPRC6A	G protein-coupled receptor family C group 6 member A
LPAR5	Lysophosphatidic acid receptor 5
MAPK	Mitogen-activated protein kinase
NPY	Neuropeptide Y
PEPT	Peptide Transporters
POMC	Proopiomelanocortin
PYY	Peptide tyrosine-tyrosine
SANRA	Scale for the quality assessment of narrative review articles
T1R	Taste receptor type 1
Y2R	Neuropeptide Y receptor type 2
